# Changes in provision of extended community pharmacy services in Aotearoa New Zealand, 2018 to 2022

**DOI:** 10.1186/s12913-024-11948-3

**Published:** 2024-12-18

**Authors:** Tara Officer, Janet McDonald, Mona Jeffreys, Jacqueline Cumming, Lynne Russell, Eileen McKinlay, Jonathan Kennedy, Ausaga Fa’asalele Tanuvasa, Caroline Morris

**Affiliations:** 1 Te Kura Tapuhi Hauora School of Nursing, Midwifery, and Health Practice, Te Herenga Waka—Victoria University of Wellington, PO Box 600, Wellington, 6140 New Zealand; 2 Te Hikuwai Rangahau Hauora—Health Services Research Centre, Te Herenga Waka—Victoria University of Wellington, PO Box 600, Wellington, 6140 New Zealand; 3 Centre for Interprofessional Education, Division of Health Sciences, University of Otago, PO Box 56, Dunedin, 9054 New Zealand; 4 Department of Primary Health Care & General Practice, Te Tari Hauora Tūmatanui, University of Otago Wellington, PO Box 7343, Wellington, 6242 New Zealand

**Keywords:** Community pharmacy services, Pharmacists, Intern pharmacists, Survey, New Zealand, Aotearoa, Health services research

## Abstract

**Background:**

Recognition is growing of the contributions community pharmacists make to the primary health care team, as their role shifts from a traditional dispensing focus to greater emphasis on fully applying their clinical skills. Some extended pharmacist services (e.g., vaccinations) are becoming increasingly common in New Zealand (Aotearoa). Community pharmacies provided key services during the COVID-19 response.

**Methods:**

An online survey was sent to community and intern pharmacists in 2022, regarding delivery of extended services and enablers and barriers to service provision, to look at changes since a 2018 survey. New questions were included regarding pharmacy services during the COVID-19 pandemic.

Descriptive analyses were conducted, and comparisons made between reported proportions in the 2018 and 2022 surveys. For questions related to attributes of pharmacies rather than pharmacists, we weighted the data by the inverse of the number of pharmacists in the pharmacy.

**Results:**

A total of 64 pharmacists and 25 interns responded to the survey. The most common services for which pharmacists were accredited were the provision of the emergency contraceptive pill (95%), sildenafil (94%), trimethoprim (88%) and immunisations (80%). There was an increase from 2018 to 2022 in the proportion of pharmacists who were accredited to provide selected oral contraceptives and immunisations. As in 2018, interns showed strong interest in gaining accreditation for all extended services. Enablers and barriers to offering extended services did not change materially between 2018 and 2022, other than a greater proportion of pharmacists reporting that they were too busy dispensing to offer more services (an increase from 46 to 63%, *P* = 0.033). Almost all (98%) reported that “COVID increased the levels of stress at work”, 94% that “workload has increased”, and 90% that “home delivery services increased.” Sixty-nine percent of pharmacists and 73% of interns reported work stress negatively affecting their wellbeing.

**Conclusions:**

Most pharmacists want to work at the top of their scope of practice and are accredited to offer a range of extended services. However, their ability to focus on more patient-facing roles continues to be constrained by the time they spend dispensing. Adequate remuneration for services provided remains a concern.

## Background

Ageing populations and the growing prevalence of chronic conditions are increasing demand on general practice and primary health care services in many countries. At the same time, widespread health care workforce shortages are making access to some services more difficult [1]. One way to support both the workload of general practitioners (GPs) and patient access is to make full use of the skills of all members of the health care team [2, 3]. There is growing recognition of the contributions community pharmacists make (including medicines management, long-term conditions management, and public health) as pharmacists shift from their traditional focus on dispensing medicines to a greater emphasis on fully applying their clinical skills, and more intensively working with patients [4–6]. The recent New Zealand Health Strategy notes the need for integrated, multi-disciplinary primary health services and “a stronger role for pharmacists and nurses in providing health advice and prescribing medication” [7]^p43^. Service users are widely reported to trust pharmacists and to be satisfied with the services they receive from a pharmacist, but some may be unaware of the extent of the services on offer [8–10].

Internationally, national service agreements and funding for community pharmacy services have also moved towards funding some clinical services, in addition to funding dispensing services. For example, the NHS England Community Pharmacy Contractual Framework 2019–2024 has funded the (i) New Medicine Service for people starting medication for certain long-term conditions, (ii) Community Pharmacist Consultation Service for minor ailments, and (iii) Discharge Medicine Service for medicines reconciliation following hospital discharge, among others [11]. Scotland has had a ‘Pharmacy First’ service for minor ailments for several years, and additional funding was announced for NHS England in 2023 for a similar service [12, 13]. In Australia, the Seventh Community Pharmacy Agreement funds several medicines management programmes (a Home Medicines Review, MedsCheck, and Diabetes MedsCheck) [14, 15]. In Aotearoa, the Integrated Community Pharmacy Services Agreement (ICPSA) includes long-term conditions (LTC) pharmacy services, immunisation services, and the Community Pharmacy Anti-coagulation Management Service (CPAMS) [16]. Such services can support medicines adherence, patient management of long-term conditions, and population health, among other things [6, 17, 18].

In Aotearoa, 76% of pharmacists work in about 1,000 community pharmacies [16, 19]. Pharmacy services were previously contracted under the ICPSA by 20 District Health Boards that were responsible for funding and/or providing services in their area. Following legislated health reforms, Te Whatu Ora – Health New Zealand became responsible for planning and commissioning health services nationally from 1 July, 2022 [20].

The ICPSA consists of nationally-consistent service schedules that are offered to all providers (dispensing and professional advisory services) and to some or all providers (including opioid substitution treatment, clozapine services and immunisation services); and locally commissioned services (including LTC, CPAMs and smoking cessation services) [16].

Some services (e.g., LTC) can be offered by any registered pharmacist, while others require accreditation through completing a training course for the specific service. The latter include CPAMS, immunisation services, and the supply of some medicines previously available by prescription only, but reclassified to permit their sale by an accredited pharmacist in specific circumstances (i.e., the emergency contraceptive pill; selected oral contraceptives; sildenafil for erectile dysfunction; and trimethoprim for urinary tract infections – superseded by nitrofurantoin in late 2022) [21–23]. Pharmacists pay for these training courses, or the cost may be covered by their employer. Training may also need to be undertaken outside of work hours. Since 2020, intern pharmacists (graduates who are completing one year of mandatory training in order to be registered as a pharmacist) have been eligible to administer influenza vaccines following training [24]. This has since broadened to COVID-19, human papillomaviruses (HPV), influenza and measles, mumps and rubella (MMR) vaccines being administered by interns; qualified pharmacists who have completed vaccination training can additionally administer herpes zoster, meningococcal and Tdap vaccines [25].

Although pharmacists in Aotearoa can now offer a growing suite of clinical services, the range offered at any particular community pharmacy varies for reasons including lack of local contracts for a service such as CPAMS or because pharmacists are not accredited. Internationally, other barriers to extended services include lack of time or insufficient funding to offer a service and the need for private consultation space within a pharmacy [26–28]. One way pharmacists can be freed from a focus on dispensing and enabled to spend more time on extended services is through effective use of other pharmacy staff, particularly Pharmacy Accuracy Checking Technicians (PACTs) [29–31]. While a pharmacist is still required to complete an initial clinical check on a prescription, this advanced technician role means the PACT can complete the final accuracy check of dispensed medicines [31]. An Australian study showed the presence of pharmacy interns also increased the provision of services in community pharmacies [32].

Some extended pharmacist services are becoming increasingly common in Aotearoa – an example is vaccination, which has widened in scope (from influenza initially, to seven vaccines currently) and in public visibility and acceptability, particularly through the contribution of pharmacists to COVID-19 vaccination campaigns [8, 33]. As part of the Government’s 2023 winter preparedness plan, a Minor Ailments Service was available for four months at community pharmacies in selected areas (where Emergency Departments are experiencing particular health service pressures), with targeted eligibility (i.e., Māori, Pacific peoples, children aged under 14 years, and people with lower incomes who hold a community service card) [34]. Conditions covered included acute diarrhoea, eye infections, scabies, headlice, dermatitis and minor skin infections [34]. Pharmacists were able to provide appropriate medicines and treatment aids to service users, free of charge, and were reimbursed through a service fee and for the cost of medicines and aids [34]. Subsequent and prior to this, regional minor ailment schemes were introduced to support specific health districts [35–37].

Community pharmacies have been a key health service provider during the COVID-19 pandemic. Their contributions in Aotearoa have varied over time, but have included continuing dispensing (pivoting to receiving more prescriptions through the e-prescribing system and by email and moving to monthly rather than three-monthly dispensing for several months in 2020); continuing to provide general health advice and over-the-counter medication; contributing significantly to the COVID-19 vaccination programme and continuing influenza vaccination; helping people to obtain a vaccine pass; supervising Rapid Antigen Testing (RAT) by unvaccinated people who required proof of a negative test for travel within Aotearoa or to attend court; providing supplies of RATs and masks; and supplying antiviral medication for eligible patients [38–44]. Pharmacies also had to make changes to operate safely in the COVID-19 environment, such as screening patients before they entered, splitting staff into different shifts, working extended hours, obtaining personal protective equipment for staff, and managing many more interactions with customers by phone [43, 45, 46].

In 2018, we surveyed all pharmacists and intern pharmacists working in community pharmacies in Aotearoa, describing the delivery of extended services and enablers and barriers to service provision at that time [47]. In 2022, we repeated this survey with the aim of exploring changes in the intervening four years, a period, as noted, that included significant demand on pharmacy services during the COVID-19 pandemic. Results from the 2022 survey are reported in this paper and compared with the findings from 2018.

## Methods

The study involved an online survey, with most questions based on our previous work four years earlier [47]. As in 2018, topics covered pharmacy ownership, location and staffing; extended services currently offered or planned; enablers and barriers to offering extended services; relationships with other local health providers; and demographic questions. New questions were added about COVID-19-related work, the experience of working in a pharmacy during the pandemic and involvement in national health reforms that were in process at the time. To accommodate the new material without overburdening participants, a few questions were removed (e.g., opening days and hours).

The survey was developed in Qualtrics [48], retaining the same wording as in 2018, as much as possible. Drafts were tested by the research team, and refinements were subsequently made to improve clarity and flow. The survey was also piloted by two pharmacists and final changes were made in response to their feedback.

The survey was available from late May to late August 2022. All pharmacists and intern pharmacists working in a community pharmacy and those working in other primary health care settings were eligible to participate; results for those working outside community pharmacy are are reported elsewhere [49]. An invitation that included an electronic link to the survey was distributed through the Pharmacy Council of New Zealand (PCNZ; the registration body for all pharmacists and interns) and the Pharmaceutical Society of New Zealand (PSNZ; a professional association, membership of which is voluntary). Both PCNZ and PSNZ published an initial notification about the survey in their newsletters and PSNZ sent out three further reminders. In addition, the survey was advertised in pharmacy media and to other groups of pharmacists to try and recruit as widely as possible.

Ethical approval for the research was granted by the Human Ethics Committee of Te Herenga Waka–Victoria University of Wellington (reference 0000030080). Informed consent was obtained at the start of the survey. On completion of the survey, participants were offered the opportunity to enter a prize draw to win one of five $100 gift vouchers.

The survey began by asking the participant’s scope of practice (i.e., pharmacist or intern – the latter then routed to a subset of relevant questions) and practice setting: community pharmacy (results reported in this paper), other primary health care (reported elsewhere) or another setting such as a hospital (not eligible to continue the survey). These initial questions were compulsory, but other questions could be skipped. Most survey questions offered single or multiple response options (including use of Likert scales), and there were some free text questions.

### Statistical methods

Descriptive analyses were conducted, including numbers and percentages of people who agreed with each statement. As most questions were not compulsory to complete, there was a significant degree of missing data. Percentages were calculated excluding people who did not answer a question and those who answered “prefer not to say”. Comparisons were made between reported proportions in the 2018 and the 2022 surveys. P values from two-sided Fisher exact tests were used to determine whether the differences could be due to chance; a *P*-value of 0.05 was considered statistically significant.

Some survey questions related to attributes of pharmacies rather than pharmacists. Given the possibility that multiple pharmacists from the same pharmacy may have taken part, we weighted the data relating to pharmacies using the inverse of the number of pharmacists that a respondent said worked in their pharmacy to generate the relevant statistics. This is the same method that was used in the earlier report.

Univariate Poisson regression was used to determine associations between attributes of the pharmacy or pharmacist and the likelihood of offering extended services. The outcome measure was the number of extended services that the pharmacist offered, and the association was reported as incident rate ratios (iRR) and associated 95% confidence intervals (CIs).

## Results

A total of 79 registered pharmacists and 29 intern pharmacists working in a community pharmacy setting consented to and began the survey; 15 pharmacists and four interns did not answer any questions beyond describing their role, so were excluded from all analyses, meaning that the main sample was based on 64 pharmacists and 25 interns. A further nine pharmacists and three interns exited from the survey before answering the questions on enablers and barriers to the provision of extended services and were excluded from those analyses.

As at 30 June, 2022, there were 3,037 practising pharmacists whose primary work was in community pharmacy [19], giving a response rate of 2%. Of the 2022 intern year group, 176 worked in a community pharmacy (S. Renner, PSNZ, Personal Communication), giving a response rate of 14%. These response rates compare with 19% of community pharmacists and 26% of interns in the 2018 survey.

The demographic distribution of respondents is shown in Table 1. About three quarters of pharmacists and interns were female; the sample of registered pharmacists were spread across age bands, including 9% aged 65 years or older. Most pharmacists were of New Zealand European ethnicity (61%) while half the interns were people of Chinese and “Other” ethnicities. There were few Indigenous pharmacists or interns, and none of the respondents identified as Pacific peoples. The participants’ demographics reflected the profession nationally in terms of gender and age, but there were more New Zealand European pharmacist respondents and a low response from both Māori and Pacific pharmacists, groups known to be under-represented in the profession relative to population proportion and health needs [19].

**Table 1:** Demographics of survey respondents in 2022

	Pharmacists (*n* = 64)			Interns (*n* = 25)		
	*n*	%	All pharmacists ^a^	*n*	%	All interns ^b^
Gender						
Female	40	74%	67%	15	71%	69%
Male	14	26%	33%	6	29%	30%
Prefer not to say / did not answer	10			4		< 1%
Age Group (years)						
< 25	7	13%	4%	19	90%	85%
25 to 34	17	31%	37%	25 or over: 2	10%	
35 to 44	5	9%	24%			
45 to 54	12	22%	16%			
55 to 64	8	15%	14%			
65 to 74	5	9%				
Prefer not to say / did not answer	10			4		
Prioritised ethnicity						
Māori	1	2%	2.1%	2	10%	Not available
Chinese	6	12%	Asian: 35.2%	4	20%	
Indian	5	10%	Middle Eastern/Latin American/African/Indian: 4.9%	1	5%	
NZ European	31	61%	48.3%	7	35%	
Other	8	16%	8.6%	6	30%	
Pacific	0	0%	0.8%	0		
Missing / prefer not to say	13			5		
Years practising as a registered pharmacist						
0 to < 5	14	26%		N/A		
5 to < 10	6	11%				
10 to < 20	9	17%				
20 to < 30	9	17%				
30 to < 40	10	19%				
40 +	6	11%				
Did not answer	10					
Likely to leave pharmacy in next 5 years						
Yes	6	11%		3	14%	
No	48	89%		19	86%	
Did not answer	10			3		

Percentages are calculated having omitted the missing data

^a^ Pharmacy Council of New Zealand. (2022). Pharmacy workforce demographic 2022. Retrieved from http://pharmacycouncil.org.nz/wp-content/uploads/2022/10/Workforce-Demographic-Report-2022-Final.pdf

^b^ S. Renner, PSNZ, Personal communication

A quarter of pharmacists had been practising for less than five years. About one in ten of the pharmacists and interns reported that they were likely to leave pharmacy in the next five years; this was a smaller proportion than we reported in 2018, at which point about one in three registered and intern pharmacists reported this likelihood.

Almost three quarters of pharmacists reported working in one of the five main centres or another large urban area (Table 2) and half were located within or near a health centre. Few respondents worked in pharmacies that were either part of a Māori health provider or served a marae (Māori cultural complex usually on tribal land) community. Similarly, few worked in pharmacies that were located with Pacific providers and communities. About three quarters of respondents reported working in an independently-owned pharmacy, and about half worked in a pharmacy that was part of a branded pharmacy group. Among the small number of pharmacy owners and managers who reported on their business’s financial sustainability, none reported that it was thriving, and about half reported that it was unable to survive in the long-term.

**Table 2:** Characteristics of the Community Pharmacies, 2022 respondents

	Pharmacists (*n* = 64)	
	n	%
Region		
Main Centre	28	57%
Other urban area (pop > 30,000)	10	15%
Urban area (pop 10,000 to 29,999)	9	13%
Small urban/rural area (< 10,000)	7	14%
Did not answer	10	
Location		
Within or next door to a health centre	32	49%
In a central city or town shopping area	19	25%
In a suburban shopping area	23	35%
Supermarket	7	5%
Part of Māori health service or in close proximity to a Māori health service/ provider and/or marae community	5	7%
Part of Pacific health service or in close proximity to a Pacific health service/ provider and/or Pacific community	3	3%
Other	2	4%
Did not answer	9	
Ownership		
Corporate	17	25%
Independent	44	72%
Other	3	3%
Part of a branded pharmacy group		
Yes	34	45%
No	30	55%
Financial sustainability		
Thriving	0	0%
Getting by	9	52%
Unable to survive long-term	7	48%
Don’t know or did not answer	2	

Based on responses from registered pharmacists only. Proportions are weighted by the inverse of the number of pharmacists in the pharmacy. The financial sustainability question was asked only of pharmacists who were pharmacy owners or managers

Pharmacists were asked which of a specified range of extended services they currently offered or were intending to get accreditation to offer in the future (Table 3). The most common services for which they were currently accredited were the provision of the emergency contraceptive pill (95%), sildenafil (94%), trimethoprim (88%) and immunisations (80%). Forty one (80%) of vaccine-accredited pharmacists had administered COVID-19 vaccines. There was strong evidence for an increase from 2018 to 2022 in the proportion of pharmacists who were accredited to provide selected oral contraceptives and immunisation, and some evidence to suggest an increase in sildenafil provision and medicine therapy assessments. Statistical tests showed that the observed increases in provision of anti-coagulation management and other services could not exclude the possibility of a chance finding. Areas in which pharmacists reported most interest in becoming accredited or authorised to offer in future (data not shown in table) included provision of selected oral contraceptives (69% of those not already accredited), trimethoprim provision (63%), and melatonin provision (62%). Fewer than 40% of pharmacists not already accredited were planning to gain accreditation for any of the other listed services.

**Table 3:** Proportion of pharmacists accredited/ authorised to offer extended services, reported in 2018 and 2022

Service	2022 Survey		2018 Survey [47]	Change	*P* value
	n	%	%		
Emergency contraceptive pill	61	95%	95%	﻿–	0.59
Sildenafil	60	94%	86%	﻿–	0.052
Trimethoprim	56	88%	85%	﻿–	0.38
Immunisation	51	80%	34%	↑	< 0.001
Selected oral contraceptives	48	75%	44%	↑	< 0.001
Melatonin	30	47%	Not asked	*	n/a
Anti-coagulation management service	19	30%	23%	﻿–	0.15
Medicines use review	16	25%	23%	﻿–	0.40
Medicines therapy assessment	6	9%	3%	↑	0.020
Other	14	22%	15%	﻿–	0.10

﻿– No change noted (either less than a 5 percentage point change from 2018 to 2022 and/or *P* > 0.05)

Percentages were calculated as the percentage of people who said yes, divided by total number of respondents, minus the number who did not say they were currently accredited (no and did not answer)

^*^ Melatonin was reclassified for supply by pharmacists on 24 June, 2019 [50]

There were no material differences between 2018 and 2022 in the extended services interns planned to become accredited to offer (Table 4). Of note, vaccination accreditation can now be achieved during a pharmacist’s intern year [24]. Twenty two of the 25 (88%) interns reported that they were already accredited, and the other three were planning to become accredited in the future. Interns reported having given vaccinations against influenza (*n* = 22, 100%), COVID-19 (*n* = 14, 64%) and MMR (*n* = 7, 32%). None had given HPV vaccines.

**Table 4:** Extended services for which intern pharmacists plan to become accredited or authorised in future, reported in 2018 and 2022

Service	2022 Survey (*n* = 25)		2018 Survey (*n* = 59)	Change	*P* value
	n	%	%		
Immunisation	3	100%	82%	*	0.11
Emergency contraceptive pill	23	92%	95%	–	0.63
Trimethoprim	23	92%	91%	–	> 0.99
Selected oral contraceptives	23	92%	91%	–	> 0.99
Sildenafil	21	84%	78%	﻿–	0.77
Melatonin	19	76%		**	n/a
Anti-coagulation management service	18	72%	75%	–	0.79
Medicines use review	16	64%	77%	﻿–	0.29
Medicines therapy assessment	15	60%	65%	﻿–	0.81
Other	4	16%	12%	–	0.73

﻿– No change noted (either less than a 5 percentage point change from 2018 to 2022 and/or *P* > 0.05)

Percentages were calculated as the percentage of people who said yes, divided by total number of respondents, minus the number who did not say they were currently accredited (no and did not answer)

^*^ Since 2020, interns have been able to complete immunisation training during their internship year

^**^ Melatonin was reclassified for supply by pharmacists on 24 June, 2019 [50]

In addition to the list of accredited services shown in Table 3, pharmacists were asked about other extended services provided at their pharmacy. The two most commonly provided services were supply of nicotine replacement therapy (91%) and blood pressure checks (80%). Again, there were few material changes of note (Table 5), other than a significant increase in the proportion of pharmacies with a clozapine or a vitamin B12 service. In addition to those shown in Table 5, further services for which fewer than five pharmacists (6% of respondents or lower) reported providing were: formal, funded selective serotonin reuptake inhibitor (SSRI) counselling services and other mental health services; various screening tests (cholesterol, bone density, Group A streptococcus, HbA1c, iron); a pain management service commissioned by the Accident Compensation Commission; and publicly-funded skin ailment services (e.g., for scabies).

**Table 5:** Other services currently provided by the pharmacy, reported in 2018 and 2022

Service	2022 Survey		2018 Survey	Change	*P* value
	n	%	%		
Supply of nicotine replacement therapy	58	91%	90%	﻿–	> 0.99
Blood pressure	51	80%	81%	﻿–	0.87
Smoking cessation counselling	48	75%	74%	﻿–	> 0.99
Gout management (CPGMS)	48	75%	Not asked		
Opioid substitution therapy	45	70%	68%	﻿–	0.78
Pharmacy clozapine services	44	69%	54%	↑	0.024
Vitamin B12	24	38%	10%	↑	< 0.001
Blood glucose	13	20%	23%	﻿–	0.75
Aseptic services, including syringe driver preparation	5	8%	Not asked		

﻿– No change noted (either less than a 5 percentage point change from 2018 to 2022 and/or *P* > 0.05)

The next part of the survey investigated enablers and barriers to offering extended services, summarised in Table 6. In general, attitudes did not change materially between 2018 and 2022, other than a greater proportion of pharmacists reporting that they were too busy dispensing to offer more services (an increase from 46 to 63%, *P* = 0.033). The proportion of pharmacists that reported needing additional training decreased significantly, whereas the proportion of owner/ manager pharmacists who felt that they had sufficient business skills and financial knowledge to manage and develop their business increased between the two surveys (37% to 59%, *P* = 0.005). There were no material changes in the reported need for additional resources or infrastructure. Consumer demand appeared to have increased markedly (64% to 84%, *P* = 0.003), and relationships between community pharmacists and other health care providers appears to have improved, with fewer pharmacists reporting concern about the impact on these relationships if they offered more services (38% to 20%, *P* = 0.008).

**Table 6:** Proportion of people who agreed or strongly agreed with each enabler or barrier to offering extended services among pharmacists, reported in 2018 and 2022

	2022 Survey (*n* = 55)	2018 Survey (*n* = 546)	Change	*P* value
Attitudes and support for extended services				
I want to work at the top of my scope of practice	91%	83%	﻿–	0.18
The pharmacy owner supports providing more services	71%	70%	﻿–	> 0.99
The pharmacy management supports providing more services	69%	68%	﻿–	> 0.99
I am excited by the new opportunities in community pharmacy	65%	56%	﻿–	0.20
I’m too busy dispensing to offer more services	63%	46%	↑	0.033
I’m happy with my current work roles and don’t want to provide more services	22%	20%	﻿–	0.73
Skills and Training				
I need additional training in order to offer additional services	64%	86%	↓	< 0.001
I feel culturally competent to deliver pharmacy services	80%	80%	﻿–	> 0.99
My employer provides funding for me to undertake training and accreditation	52%	57%	﻿–	0.40
My employer allows me time to undertake training	34%	**		
I can afford training and accreditation	53%	42%	﻿–	0.15
I have the business skills and financial knowledge to manage and develop my business in the current environment *	59%	37%	↑	0.005
My employer allows me time to sit accreditation assessments	35%	***		
Resources and infrastructure				
The pharmacy has a suitable private consultation area to talk with people confidentially	82%	82%	﻿–	> 0.99
The pharmacy has enough equipment or other resources to enable it to provide more services (e.g. fridge space to store vaccinations)	76%	65%	﻿–	0.10
I have access to relevant patient health information from other health providers	60%	54%	﻿–	0.48
The pharmacy can access funding for more services	33%	25%	﻿–	0.74
There are sufficient technicians or other support staff to free up my time to offer more services	14%	24%	–﻿	0.13
The available funding covers the cost of providing the service	15%	11%	–﻿	0.38
Consumers and local relationships				
There is consumer demand for more services	84%	64%	↑	0.003
I’m concerned about the impact on my relationships with other health providers if I provide more services	20%	38%	↓	0.008
Consumers can afford to pay for additional services	24%	30%	–﻿	0.36
Consumers are willing to pay for additional services	15%	26%	–﻿	0.071

– No change noted (either less than a 5 percentage point change from 2018 to 2022 and/or *P* > 0.05)

^*^ restricted to owner/managers (*n* = 17)

^**^ Asked slightly differently in earlier study, so a comparison is not valid

^***^ Not asked in earlier study

The associations between the presence of enablers/ barriers and the number of extended services offered is shown in Table 7. None of these associations reached conventional levels of statistical significance, which is likely to be due to the small sample size. It could also be that factors other than those measured determine whether or not practices offer more extended services.

**Table 7:** Unadjusted Poisson regression analyses of the association between attributes of the pharmacy or pharmacist and the likelihood of offering extended services, 2022

	RR	CI
Attitudes and support for extended services		
I want to work at the top of my scope of practice	1.19	(0.76, 1.87)
The pharmacy owner supports providing more services	1.09	(0.84, 1.41)
The pharmacy management supports providing more services	1.10	(0.85, 1.41)
I am excited by the new opportunities in community pharmacy	1.17	(0.92, 1.48)
I’m too busy dispensing to offer more services	0.94	(0.75, 1.18)
I’m happy with my current work roles and don’t want to provide more services	0.91	(0.69, 1.19)
Skills and Training		
I need additional training in order to offer additional services	0.87	(0.70, 1.09)
I feel culturally competent to deliver pharmacy services	0.90	(0.68, 1.18)
My employer provides funding for me to undertake training and accreditation	1.07	(0.86, 1.34)
My employer allows me time to undertake training	1.13	(0.90, 1.42)
I can afford training and accreditation	1.02	(0.82, 1.27)
I have the business skills and financial knowledge to manage and develop my business in the current environment	0.96	(0.63, 1.46)
My employer allows me time to sit accreditation assessments	1.10	(0.87, 1.38)
Resources and infrastructure		
The pharmacy has a suitable private consultation area to talk with people confidentially	1.15	(0.85, 1.55)
The pharmacy has enough equipment or other resources to enable it to provide more services (e.g. fridge space to store vaccinations)	1.15	(0.88, 1.51)
I have access to relevant patient health information from other health providers	0.90	(0.72, 1.12)
The pharmacy can access funding for more services	1.03	(0.78, 1.37)
There are sufficient technicians or other support staff to free up my time to offer more services	1.14	(0.84, 1.53)
The available funding covers the cost of providing the service	0.89	(0.64, 1.24)
Consumers and local relationships		
There is consumer demand for more services	0.97	(0.66, 1.41)
I’m concerned about the impact on my relationships with other health providers if I provide more services	1.15	(0.88, 1.49)
Consumers can afford to pay for additional services	0.84	(0.64, 1.11)
Consumers are willing to pay for additional services	0.93	(0.68, 1.28)

Pharmacists’ and interns’ experiences of working through the acute COVID-19 response are shown in Table 8. Overall, the proportion of pharmacists who agreed with the positive statements was relatively low; 70% reporting more people seeking clinical advice via telehealth methods, 67% feeling safe at work in the COVID-19 environment, and 62% agreeing email (and 60% e-prescription) processes worked well. Agreement with positive staffing statements was low, with about one in five agreeing that there was sufficient staffing, and a similar number reporting that finding staff to support the COVID-19 response was easy. In general, interns reported similar experiences. In contrast, many more pharmacists and interns agreed with the negative statements, with almost all pharmacists (98%) reporting that “COVID increased the levels of stress at work”, 94% reporting that workload has increased, and 90% reporting that home delivery services had increased. Worryingly, 69% of pharmacists and 73% of interns reported work stress negatively impacting their wellbeing.

**Table 8:** Proportion of people who agreed or strongly agreed with statements relating to the COVID-19 response

	Pharmacists (*n* = 55)	Interns (*n* = 22)
Positive experiences		
More people are seeking clinical advice by phone/text/email etc	70%	–﻿
I feel safe working at this pharmacy in the COVID environment	67%	73%
The email prescription process works well^a^	62%	68%
The e-prescription process works well^a^	60%	91%
Providing services outside the walls of the pharmacy has increased	54%	–﻿
Managing Rapid Antigen Testing (RAT) processes is straightforward	50%	45%
It was easy to set the pharmacy up to operate safely^b^	47%	–﻿
Communication with other health professionals has improved	24%	
There is sufficient staffing to cater to service-user needs	19%	23%
Finding staff to support the COVID-19 response has been easy^b^	19%	﻿–﻿
Negative experiences		
COVID increased the levels of stress at work	98%	82%
My workload has increased	94%	﻿–﻿
Home delivery services increased	90%	﻿–﻿
Work stress is negatively impacting my wellbeing	69%	73%
COVID has reduced the viability of this pharmacy^b^	50%	﻿–﻿

^a^ Only asked of pharmacists (*n* = 52) and interns (*n* = 22) who stated that they had an e-prescription or e mail prescription service

^b^ Only asked of the 18 pharmacist owners or managers

Pharmacists reported spending the majority of their time (70%) on dispensing (see Table 9). Other activities that took significant time included offering extended services (15%), COVID-19-related work, including COVID-19 immunisations (13%), and administration (10%). The pattern for interns was a bit different; they tended to spend less time on dispensing (57%) and more time on administering immunisations (26%) and other clinical patient-facing work (20%), with a similar amount of time on COVID-19-related work (15%) and administration (10%).

**Table 9:** Approximate percentage of time spent on each activity, as reported in 2022

Activity	Pharmacists	Interns
Dispensing	70% (50 to 84)	57% (46 to 65)
Extended services	15% (7 to 26)	10% (4 to 18)
COVID-19-related work	13% (5 to 23)	15% (11 to 27)
Administration	10% (5 to 24)	10% (3 to 25)
Other clinical patient-facing work	7% (4 to 21)	20% (10 to 26)
Immunisations, excluding COVID	6% (3 to 24)	26% (9 to 34)

Figures are given as median percentages (inter-quartile range)

## Discussion

As in 2018, our results demonstrate wide variation in the services pharmacists were accredited to offer. In 2022, the three most common accredited services were provision of the emergency contraceptive pill, sildenafil and trimethoprim, with only sildenafil showing some evidence of increased provision compared with 2018. (Subsequent to the survey, trimethoprim has now been replaced by nitrofurantoin as first-line treatment for uncomplicated urinary tract infections [23]). Between 2018 and 2022, immunisation accreditation significantly increased from 34 to 80%, likely influenced by pharmacists’ role in COVID-19 vaccination as the pandemic response necessitated better utilisation of the whole health workforce [43]. Acceptance of pharmacist vaccinators is also reflected in the greater range of immunisations now available and funded at community pharmacies. Provision of selected oral contraceptives also showed a significant increase since 2018 (from 44 to 75%), and there was strong interest in future accreditation, reflecting what was a relatively new service in 2018 is becoming embedded in pharmacists’ practice. Similarly, provision of melatonin had uptake by almost half the pharmacists, with 62% of the remainder interested in offering this service in the future. Melatonin provision by pharmacists has only been available since 2019, for treating primary insomnia in people aged 55 or older [50]. In contrast, 30% or fewer pharmacists were accredited for any of the other services and nor was there much future interest. This may reflect barriers to uptake of these services, such as the need for a local contract to offer CPAMS and general lack of funding for medicines use review or medicines therapy assessment. As well as accredited services, a range of other services were being provided by pharmacies, with little change since 2018, other than 75% offering the community pharmacy gout management service (a new question in the 2022 survey).

Among interns, there was strong interest in becoming accredited for all extended services in future, once they are legally able to do so, and this had not changed from 2018. There was least interest in medicines therapy assessment (60%) and medicines use review (64%), but these are still markedly higher than pharmacists’ reported future interest. This may reflect some lack of knowledge among interns about the reality of offering services (such as whether contracts or funding will be available). However, it also signals expectations that their role will have a focus on extended clinical services. Most were already accredited vaccinators as this is now possible during their intern year, and the remainder planned to become accredited, indicating this role will be well-embedded during their continuing careers.

Table 6 suggests some reasons behind why extended services may or may not be offered. Most (91%) pharmacists indicated they wanted to work at the top of their scope of practice, and compared with 2018, fewer said they needed additional training to offer additional services. However, 63% said they were too busy dispensing to offer more services, a significant increase from 2018. In addition, few (14%) said there were sufficient technicians or other support staff to free up their time to offer more services. Pharmacists need to be able to move away from the dispensary if they are to offer more extended services, but are still spending the majority (70%) of their time dispensing.

PACTs are an important enabler of moves away from pharmacist-dispensing roles into patient-facing roles [30, 51, 52]. Yet, technician role development may be limited if pharmacists are not confident in advanced technician roles [53] and if technicians do not see tangible benefits, such as increased pay, if they undertake further training [54, 55]. In other research, some technicians felt cost and some entry requirements were barriers to joining the PACT training programme in Aotearoa, leading to a review of the programme and changes to the governance, operational and training frameworks in 2022 [56, 57]. As of July 2023, there were 148 practicing PACTs in Aotearoa and a further 58 in training (K. Rodgers, PSNZ, personal communication). Time and staffing are oft-reported barriers to offering additional extended services, both in Aotearoa and internationally [26, 27, 58, 59].

Another factor that may be limiting growth of extended services is funding, with only 15% of pharmacists agreeing that available funding covered the cost of providing the extended service. Notably, service contracts such as the ICPSA are with individual pharmacies, and not pharmacists. Inadequate remuneration has been widely noted previously as a barrier to extended service provision [26, 59]. The recent new Minor Ailments Service ran for a period of four months during the winter but only in restricted areas, and for a limited group of patients. While it funded pharmacists for their consultations and any approved medicines and treatment aids that they supply [34], the move towards more district-based services highlights regional discrepancies in the ability to provide extended services [34]. Notably, the success of the Minor Ailments Service means Te Whatu Ora – Health New Zealand are considering running an enduring service [60].

COVID-19 has provided an opportunity for community pharmacy to contribute in many ways, but the survey responses indicate significant additional workload and stress. An Australian survey of pharmacists’ experiences during the COVID-19 pandemic reported high burnout, and an interview study with Australian pharmacists also described increased pressures and reduced mental wellbeing [61, 62]. In a post-pandemic environment, where arguably pharmacy service requirements have not eased, having an understanding of how pharmacy contributes to service delivery is timely and can inform future health service planning.

Two important areas of change highlighted by the survey findings were a marked increase in agreement that there is consumer demand for more services, and a significant decrease in reported concern about the impact on a pharmacist’s relationships with other health providers if they offered more services. The former may signal increased consumer awareness of pharmacists’ skills and services, likely aided by the continued accessibility of pharmacy services during the pandemic and pharmacists’ involvement in COVID-19 vaccination. The latter may indicate a growing acceptance of extended pharmacist roles by other health professionals, in contrast to some earlier research that identified GPs’ attitudes and lack of collaboration as impeding pharmacists’ service development [63–65]. A recent Australian survey of extended services, however, also identified less concern about relationships between pharmacists and GPs over time [26].

### Limitations

The greatest limitation of this work is the low response rate to the 2022 survey. Although we used a number of methods to attempt to increase this, such as having three reminders sent out by the PSNZ and timing the survey to not coincide with annual practicing certificate renewal, community pharmacists have been incredibly busy since the COVID-19 pandemic began. Early in the pandemic (March-July 2020), regular prescriptions could only be dispensed for one month’s supply instead of the usual maximum of three months, in an attempt to avoid supply chain disruptions [44]. This meant that pharmacists’ dispensing workload increased significantly during that period [66]. As the pandemic evolved, pharmacies were an important first line source of primary health care services [67]. Furthermore, pharmacists provided much of the COVID-19 and influenza vaccination workforce, meaning that 2021 and 2022 were a particularly busy time [33, 38]. Our low response rate means that some of the questions asked are open to the possibility of selection bias if respondents were more or less likely to agree with some of the enabler or barrier questions than non-respondents would have been. Nevertheless, we believe that the data provide an important insight into an under-researched but key primary health care workforce. In addition, having the ability to compare the 2022 results to those from 2018 gives important insights into changing attitudes and issues at a time of unprecedented challenges for the pharmacy workforce. Given the lack of national reporting around accreditation for the delivery of extended services, our findings also offer a timely indication of future accreditation trends in the profession.

## Conclusions

Most pharmacists want to work at the top of their scope of practice and are accredited to offer a range of extended services. However, their ability to focus on more patient-facing roles continues to be constrained by the time they spend dispensing, in the absence of sufficient technicians to free up their time. Adequate remuneration of services also remains a concern. Although pharmacists have contributed significantly throughout the COVID-19 pandemic, the additional workload has also come with stress for many. While we have highlighted some developments in extended services between 2018 and 2022, if pharmacists working at top of scope is to become standard practice [68], health policy needs to support this by funding new services appropriately and supporting the development of technician roles.

## Data Availability

The data sets generated and/or analysed during the current study are not publicly available as ethics approval was given on condition that only members of the research team would have access to the data.
